# Cold-Cured Bisphenolic Epoxy Adhesive Filled with Low Amounts of CaCO_3_: Effect of the Filler on the Durability to Aqueous Environments

**DOI:** 10.3390/ma14061324

**Published:** 2021-03-10

**Authors:** Anna Rudawska, Mariaenrica Frigione

**Affiliations:** 1 Faculty of Mechanical Engineering, Lublin University of Technology, Nadbystrzycka 36 St., 20-618 Lublin, Poland; 2 Department of Innovation Engineering, University of Salento, Via Arnesano, 73100 Lecce, Italy; mariaenrica.frigione@unisalento.it

**Keywords:** aqueous environment, calcium carbonate, durability, epoxy resin, mechanical properties, compressive modulus, compressive strength

## Abstract

The effects of aging exposures to three non-saline aqueous environments on the compressive mechanical properties of a calcium carbonate-filled bisphenolic epoxy adhesive, cold-cured with the addition of two curing agents suitable for the cure at ambient temperature (i.e., Mannich base and triethylenetetramine), were assessed. The amount of the added filler (CaCO_3_) varied from 1 to 3 g per 100 g of resin; the immersion times in each of the selected medium varied from 1 to 10 months. It was found that the mechanical properties measured in compression mode on cylindrical specimens of unfilled and CaCO_3_-loaded epoxy were scarcely influenced by the kind of curing agent employed; only the compressive modulus was limitedly affected by this parameter. Referring to the behavior when aged in water, the CaCO_3_-filled epoxies displayed noticeable growths in modulus, small reductions in strength, and limited variations in strain, with a certain influence of the exposure time, especially when comparing the properties at the lowest time with those at medium–long times. On the basis of the results of statistical MANOVA analysis, it can be concluded that among the compositional factors (i.e., the type of curing agent employed to cure the epoxy compounds and the micro-filler content), only the amount of CaCO_3_ filler significantly affects the compressive modulus.

## 1. Introduction

Epoxy resins are commonly employed in an extensive variety of different applications as structural adhesives being characterized by a mix of advantageous outstanding properties, such as thermal stability and chemical resistance, high mechanical performance, and the capability to join very different (organic, inorganic, metallic) materials [1]. Therefore, their features are conveniently exploited in the manufacture/production of coatings for metals, matrices for fiber-reinforced composites, electric insulators, electronic components, and decorative materials for flooring applications [2]. The addition of proper fillers can widen the applicability of epoxies in other fields and for more demanding applications [3].

The introduction of the inorganic fillers is a common practice to adjust one or more properties of a polymeric matrix, not least to reduce its cost. The chemical nature and amount of added filler; its size, shape, and aspect ratio; its morphology; its degree of dispersion, and interactions with the polymer are the parameters mainly affecting the effects produced on the resin matrix, including its physical/mechanical properties and durability [4,5,6]. 

Although the use of nano-sized fillers has widely developed in recent years with the aim of providing the resins with new functions and improved performance [7,8,9,10,11], the use of microscopic fillers in epoxies still deserves attention and analysis, as these particles can give the resins improved properties at very low prices compared to the much more expensive nano-fillers. Moreover, the incorporation of micro-fillers into the resin involves fewer technological problems than the inclusion of the nano-particles, such as the achievement of a homogeneous dispersion of the nano-phases in the epoxy matrix: this aspect represents a further advantage.

Calcium carbonate (CaCO_3_) is commonly added in different shapes (round particles, rods, platelets) to polymers as the inorganic filler possessing many favorable features, such as ease of being incorporated in macromolecular materials, low environmental impact and toxicity, wide availability, and cheapness. This low-cost filler in micrometric size is able to improve the characteristics of epoxy coatings and paints (i.e., hardness, scratch resistance, corrosion resistance, thermal stability, Young modulus), as well as the performance of epoxy-based adhesives and composites (i.e., tensile and flexural strengths, impact strength and toughness, wear resistance), to enhance the flashover voltage properties of epoxy intended for electrical applications [3,5,12,13,14,15,16,17,18,19,20,21].

Generally speaking, the transformation from a liquid resin to a hard, rigid, and resistant epoxy occurs by means of a chemical reaction, i.e., the cross-linking process, upon the addition to the resin of a wide range of curing agents, the more frequently used being polyfunctional amines and acid anhydrides. A distinctive advantage of the epoxy resins resides in their possibility to be cross-linked in any condition of temperature, ranging from ambient (even close to 0 °C) temperatures, as in the case of “cold-curing” epoxies applied on field as structural adhesives, to very high temperatures (i.e., around 180–200 °C) for more demanding applications, for instance in the manufacture of high-performance composites based on heat-cured epoxy resin matrices. Depending on the application, and in turn on the temperature at which the resin must set and harden, a proper curing agent must be selected. The chemical nature and the composition of the epoxy/curing agent couple significantly affect the final properties and performance of the cured resins, as well as their long-term durability under service conditions. On the other hand, this latter characteristic also depends in a complex way on the environmental exposure and load regime [22,23,24]. The mechanical, adhesive, and functional properties of epoxies can be appreciably impaired due to aging. The obtainment of the structural resins with suitable mechanical properties that are stable over time, even if exposed to adverse environmental conditions, is an indispensable asset in many applications, such as in automotive and aeronautical ones or when the epoxies are employed to realize fiber-reinforced composites to strengthen the buildings and the infrastructures.

Referring to the environmental agents to which epoxy resins can be most frequently exposed, water is indubitably the more recurrent. Pure water can be present in liquid (rain, freshwater, water of rivers, lakes, etc.) or vapor (moisture) states; it is able to enter epoxies and to severely affect in reversible (i.e., plasticization) or irreversible (i.e., hydrolysis) ways the resin properties and load-bearing capabilities [22,25,26,27,28]. A proper modification of the epoxy resins with the introduction of suitable filler can enhance its durability, thus assuring its reliability when exposed to this very common external agent [29,30]. In fact, the fillers can physically hinder water ingress into the resin [31], or else the water absorption can be restricted by the hydrophobic nature of the filler [32].

It is reported that the calcium carbonate filler has a beneficial effect on the resistance of the epoxy resin to water [31,32,33,34]. In fact, the presence of this micro-filler, in quantities ranging from low percentages to almost 50%, leads to a lower water saturation value of the loaded resins compared to the pristine one, particularly at higher CaCO_3_ content, which is a phenomenon that is attributed to the more tortuous path that water encounters when it enters the resin in the presence of the micro-filler. The same studies also showed that the reductions in mechanical tensile properties (strength and modulus) due to plasticization of the resin were smaller than those observed for the same unfilled epoxy.

The mentioned studies, nor other studies to the best of our knowledge, did not take into consideration the compressive mechanical properties of a CaCO_3_-loaded epoxy resins exposed to aqueous environments. On the other hand, the compressive properties play an important role when epoxies are used as structural adhesives in a wide variety of applications, applications in which the resins can be eventually exposed for prolonged times to the action of water (such as, for example, when the adhesive is outdoor exposed).

In a previous study [35], the effect of non-saline aqueous environments on the compressive properties of a commercial cold-cured bisphenolic epoxy adhesive filled with low amounts of CaCO_3_ was assessed as a function of the kind of curing agent, the filler content, and the aging time. In the present study, the same experimental procedure was repeated employing a different resin commercially available as structural adhesive, i.e., a diglycidyl ether of bisphenol A epoxy modified with a polyester resin, cold-cured with the same curing agents, loaded with the same amounts of calcium carbonate micro-filler and aged to the same aging regimes. In fact, the aim of this paper was to establish if the interesting findings found in the previous study related to the effects of the filler on the mechanical behavior of a bisphenolic epoxy resin aged in water for prolonged times can be generalized for other cold-cured epoxy-based adhesives. Moreover, starting from cold-cured thermosetting adhesives displaying different mechanical properties, it is possible to identify the most suitable composition (in terms of resin/curing agent couple and filler content) for each application.

## 2. Materials and Methods

### 2.1. Materials and Production of Cold-Cured CaCO_3_-Filled Epoxy Systems

The resin object of the present study (trade name Epidian 57) was a diglycidyl ether of bisphenol A (DGEBA) epoxy modified with a polyester resin, supplied by Ciech-Sarzyna (Nowa Sarzyna, Poland). The epoxide equivalent weight of Epidian 57 is about 250 g/mol; the resin, employed as adhesive cured at ambient temperature (i.e., cold-cured) to bond different materials (e.g., metals, glass, ceramics, thermosetting plastics, leather), displays good mechanical properties, high shear and peel strengths, and a good resistance toward acids, except acetic and concentrated hydrochloric acids. Other properties and characteristics of Epidian 57 epoxy resin are reported in Table 1.

Epidian 57 epoxy resin was cold-cured upon the addition of two different curing agents, which are both suitable for the cure of epoxy resin at ambient temperature, namely TFF and Z-1. TFF is a Mannich base curing agent (commercialized by Ciech-Sarzyna, Nowa Sarzyna, Poland) displaying an amine number of 500–700 mg KOH/g. The TFF curing agent was selected for its fast reactivity with epoxy even at ambient temperature as well as being able to improve the chemical resistance and thus durability of the epoxy exposed to different environments. In Table 2, some properties and characteristics of this curing agent are reported.

The second curing agent selected to cure Epidian 57 resin at ambient temperature was a triethylenetetramine (TETA) with an amine number of 1100 mg KOH/g, again supplied by Ciech-Sarzyna Company (Nowa Sarzyna, Poland). Some properties and characteristics of the Z-1 curing agent can be found in Table 2. The TETA curing agent is an aliphatic amine, typically used to cold-cure epoxy adhesives. It is able to impart improved flexibility and good impact resistance to the resin; i.e., key mechanical properties for specific applications. Therefore, different mechanical properties of the same epoxy-based adhesive cold-cured with the two curing agents, after aging in non-salty water, may be expected.

Two control (unfilled) formulations were produced: the cold-curing Epidian 57 epoxy with the Mannich base or TETA curing agents. The compositions (expressed in weight percentages and corresponding to the stoichiometric epoxy/amine molar ratio for each curing agent) of the reference formulations were Epidian 57:TFF 100:22 (indicated as E57/TFF/100:22 system) and Epidian 57:Z-1 100:10 (indicated as E57/Z-1/100:10 system), respectively.

Starting from Epidian 57 epoxy resin, modified formulations were produced by adding different amounts of CaCO_3_ filler, i.e., 1, 2, or 3 g of the calcium carbonate per 100 g of epoxy resin; then, these epoxy mixtures were cold-cured upon the addition of two curing agents, i.e., TFF and Z-1. The compositions of the resulting epoxy compounds are reported in Table 3. The anhydrous calcium carbonate (CaCO_3_) filler, supplied in powder form by Carl Roth GmbH + Co KG (Karlsruhe, Germany) with the trade name E170, displayed a micrometric particle size, i.e., 0.87 ± 0.1 μm.

**Figure 1:**
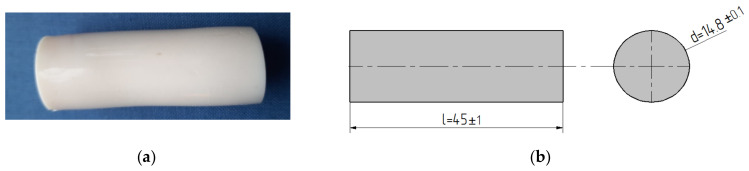
Schemes: (**a**) Cylindrical epoxy specimen after curing; (**b**) Dimensions in mm of epoxy specimen.

The detailed procedure to manufacture the CaCO_3_–epoxy systems can be found in a recently published paper [35]. After a curing time of 7 days at ambient temperature (i.e., 22 ± 1 °C, R.H. 24 ± 4%), the specimens possessing a cylindrical shape, compliant with PN-EN ISO 604-2006 code, were subjected to different tests, as described in the next paragraph. A single-stage 7-day curing time was employed, as suggested by suppliers and previously experienced in other studies [24,35,36,37,38]. After this curing time at ambient temperature, a curing degree around 95% was measured. Furthermore, this is the polymerization time used on site to obtain an almost complete polymerization of the resin with an optimal development of the mechanical and adhesive properties. For each of the compositions reported in Table 3, 6 unaged specimens were tested, plus another 6 specimens for each of the 3 aging regimes (described next) and each of the 3 immersion times. In Figure 1, one of the produced cylindrical epoxy specimens is shown, along with its dimensions.

### 2.2. Aging Regimes

The epoxy-based compounds, filled with different amounts of the CaCO_3_ and cold-cured according to the procedure described in the previous paragraph, were exposed for prolonged times to different environments (demineralized, distilled, and spring water, the latter provided by Gardinia brand (Polish Water Sp. z o.o., Aleksandria, Poland) in order to analyze the effects of aqueous media, differing for composition, on the mechanical behavior of the CaCO_3_–epoxy systems. The aim of the research was to assess if the presence of a substantially economic filler, such as calcium carbonate micro-particles, can improve the durability of any epoxy adhesive if exposed to a very common environment, such as non-saline water (present as rain/moisture, freshwater, water basins, rivers, or lakes). In a previous paper, it was demonstrated that the presence of this micro-filler is able to mitigate the plasticization effect of water on the mechanical properties, tested in compression mode, of a cold-cured epoxy based adhesive [35]: this research would demonstrate that this observation can be extended to other cold-cured CaCO_3_-filled epoxy adhesives. The time of exposure was taken as an additional parameter of the study, the immersion in each aqueous environment being performed for 1, 6, and 10 months. An overview of the accomplished aging procedures is reported in Table 4. Six specimens for each composition were subjected to each of the aging regimes/times.

### 2.3. Mechaical Tests Performed in Compression Mode

The epoxy-based compounds, unmodified or filled with different amounts of CaCO_3_, cold-cured as detailed in Section 2.1, were mechanically characterized before and after different periods of exposure to the three aqueous environments. The mechanical tests were performed at ambient temperature on cylindrical specimens (6 for each composition/type of aging regime) in compression mode according to the PN-EN ISO 604 standard [39]. A hydraulic testing machine (Zwick/Roell Z150, ZwickRoell GmbH&Co. KG, Ulm, Germany) was employed. The test parameters were initial force of 100 N; rate of 10 mm/min. The mechanical properties measured during the tests were the compressive modulus (MPa), the maximum compressive strength (MPa), and the strain at break (%).

The experimental results relative to the compressive modulus collected for each set of the composition/aging regime were subjected to a statistical analysis, eliminating the outermost results. To this aim, the ANOVA main effect analysis—the MANOVA analysis—and the Statistica 13.1 software were used. The statistical analysis was performed in two steps: the first step concerned the determination of normal distribution (at the significance level α = 0.05 using the Shapiro–Wilk test); in a second step, relevant statistical models and statistical tests were applied (e.g., assumptions about homogeneity of variance using Levene’s test). In a further step, the Tukey test was used as a post hoc test to compare statistically significant factors.

## 3. Results and Discussion

### 3.1. Effect of CaCO_3_ Filler Addition to the Unaged Epoxy Adhesives

**Figure 2:**
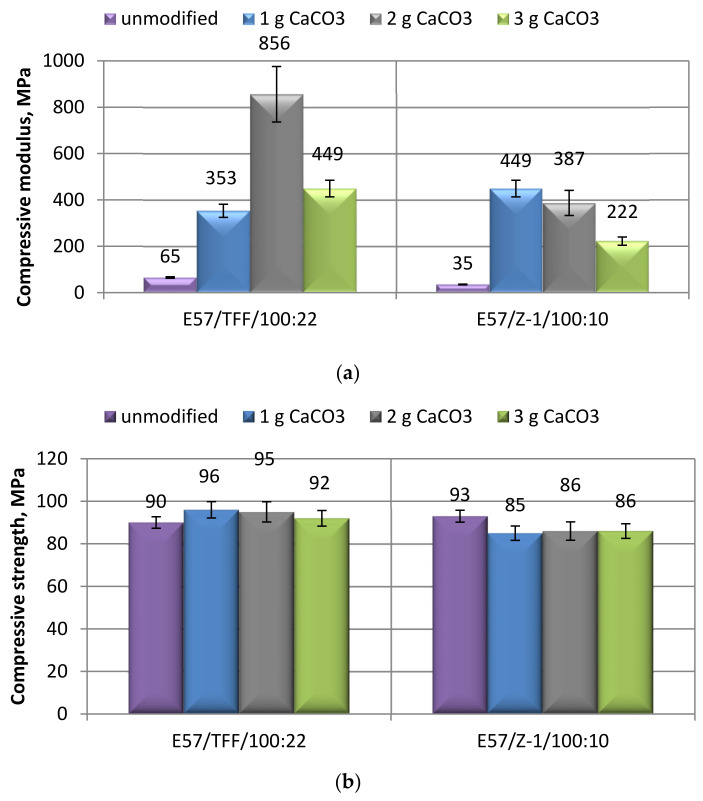

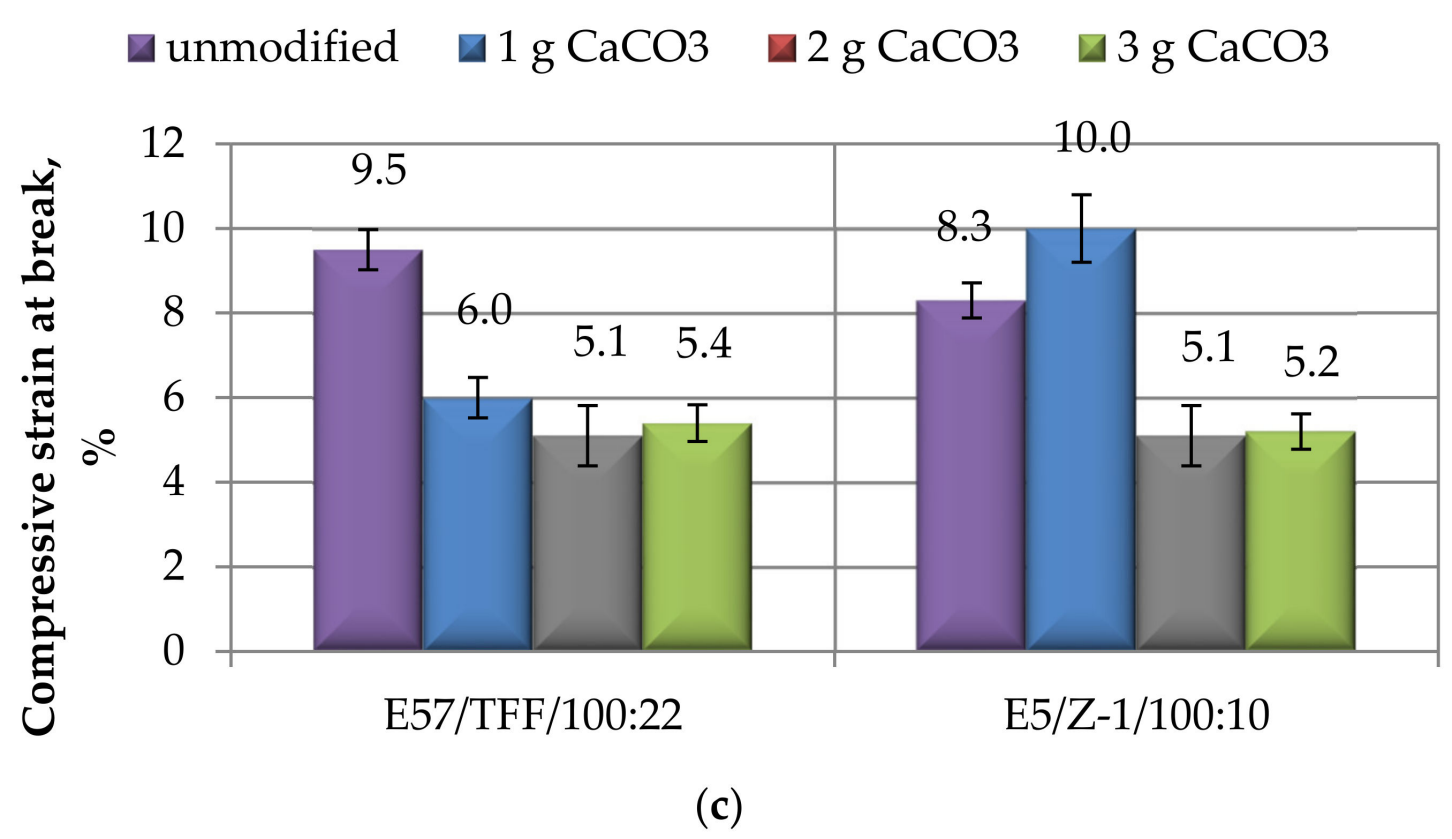
Results of mechanical tests performed in compression mode on unfilled and CaCO_3_-filled specimens, tested before any aging procedure, based on E57/TFF/100:22 and E57/Z-1/100:10 systems, respectively, in terms of: (**a**) Compressive modulus; (**b**) Compressive maximum strength; (**c**) Compressive strain at break.

The effect of the addition of small amount of calcium carbonate micro-filler on the mechanical properties of a bis-phenolic epoxy resin, i.e., Epidian 57, cold-cured with the two selected curing agents, i.e., a Mannich base and a triethylenetetramine, was first assessed. The results of mechanical tests (in terms of modulus, maximum strength, and strain at break) performed on filled and unfilled epoxies in compression mode are presented in Figure 2a–c.

Analyzing first the effect due to the different curing agents selected for the cure of the epoxy at ambient temperature, it can be said that the compressive maximum strength (Figure 2b) was scarcely influenced by the kind of curing agent employed, taking into account the variations of experimental results. An appreciable influence of the curing agent on the compressive modulus can be assessed by observing the results of Figure 2a, the resin cold-cured with the Mannich base being able to develop a double modulus compared to the same TETA amine-cured epoxy. The selection of the curing agent had a certain influence also on the strain at break measured in the compression mode, displaying the E57/TFF/100:22 system a value of this parameter somewhat greater (+12.6%) than that measured on the compound E57/Z-1/100:10. By comparing these results with those obtained on another bis-phenolic commercial epoxy adhesive (i.e., Epidian 5), possessing a lower equivalent weight (196–208 g/mol) and cold-cured in the same conditions with the same curing agents (in stoichiometric ratio) [35], it can be concluded that the E57 resin is able to display a lower compressive maximum strength (about 18–20%) and strain at break (around 21–24%), irrespective to the curing agent employed, than Epidian 5 epoxy resin. On the other hand, the effect of the curing agent on the compressive modulus of the two epoxies seems to be opposite: a greater modulus was obtained by cold-curing E57 resin upon the addition of a Mannich base curing agent, this value being much greater (+52%) than that measured on Epidian 5 cold-cured with the same curing agent; the cure with TETA curing agent was more favorable, in terms of an increased compressive modulus, for Epidian 5 resin (+66% with respect to E57 resin cold-cured with the same curing-agent). Due to the wide variety of applications in which epoxy resins are employed, the selection of the most appropriate resin/curing agent pair must be done taking into consideration the stress regime that the adhesive will have to withstand as well as the expected mechanical behavior.

The addition of CaCO_3_ filler to E57 resin brought about appreciable modifications to compressive mechanical properties: irrespective of the curing agent employed, a general increase in modulus along with a decrease in compressive strain at break were observed, the extent of the changes being influenced by the filler content. On the other hand, the compressive maximum strength remained substantially unaffected by the addition of calcium carbonate, regardless of the curing agent and the CaCO_3_ content.

By analyzing the results in depth, the addition of a small amount (2 g) of the micro-filler to the E57/TFF/100:22 system produced a marked growth in compressive modulus of over an order of magnitude (Figure 2a); even though the standard deviation value for this result is large, amounts of the same filler slightly greater or lower, i.e., 1 or 3 g, still caused appreciable increases in the compressive modulus, indicating a clear stiffening effect of the CaCO_3_ addition to E57 resin.

Similarly, the addition of calcium carbonate to the E57/Z-1/100:10 system led to noticeable increases in compressive modulus, ranging an order of magnitude with the addition of 1 or 2 g of filler (Figure 2a); in correspondence of a larger amount (3 g), the modulus is reduced, remaining still much greater than that measured on an unfilled system. Therefore, the positive effect on the compression modulus of CaCO_3_ addition to the epoxy resin under analysis is confirmed.

The observed results are in good agreement with those found by adding the same amounts of the same filler to cold-cured Epidian 5 epoxy [35], i.e., appreciable increases in compressive modulus upon the addition of small percentages of calcium carbonate. Similar results have been recently observed also when the CaCO_3_-filled epoxy was tested in tensile mode [17,31,33,34] and attributed to the much greater elastic modulus of CaCO_3_ with respect to the average modulus of an epoxy.

As already mentioned, the addition of small amounts of CaCO_3_ filler did not affect significantly the compressive maximum strength of E57 epoxy, as can be seen from the data reported in Figure 2b. Referring to the curing agent employed to cold-cure the resin, limited increases in strength (never greater than 6%) were observed employing the Mannich base curing agent; the cold-cure performed with the triethylenetetramine produced small reductions in compressive maximum strength, around 7–8%.

Starting from a more resistant resin, i.e., Epidian 5 epoxy, the addition of up to 3 g of CaCO_3_ filler produced growths in maximum compressive strength depending on the curing agent employed and on the amount of filler [35]. Therefore, the substantial invariability of this property observed in the present study upon the addition of calcium carbonate micro-filler can be attributed to the lower compressive strength value displayed by unfilled E57 resin, which probably does not allow the filler to carry out its strengthening action.

The strain at break values measured on both E57/TFF/100:22 and E57/Z-1/100:10 systems containing 2 and 3 g of calcium carbonate were distinctively lower than those measured on the respective unfilled systems (Figure 2c). The two systems only differed upon the addition of the smallest amount of the filler, i.e., 1 g, the compressive strain of E57/TFF/100:22 system being decreased (about 37%) with respect to the unfilled resin; instead, an increase in this property (of about 20%) was measured for the E57/Z-1/100:10 compound filled with 1 g of calcium carbonate. However, this latter result deserves further investigation.

These results are substantially in line with those presented in the previous mentioned study [35], where reductions in the compressive strain of cold-cured of Epidian 5 epoxy were observed upon the addition of small amounts (1 to 3 g) of CaCO_3_ filler. Moreover, these results, observed also when the CaCO_3_-filled epoxy resins are tested in tensile mode [17,31,33,34], are consistent with the increase in the elastic modulus and can be explained in terms of the stiffening and embrittlement of the epoxy as a consequence of the addition of the filler.

### 3.2. Effect of Aqueous Aging Regimes on CaCO_3_-Filled Mannich Base-Cured Epoxy Adhesives

**Figure 3:**
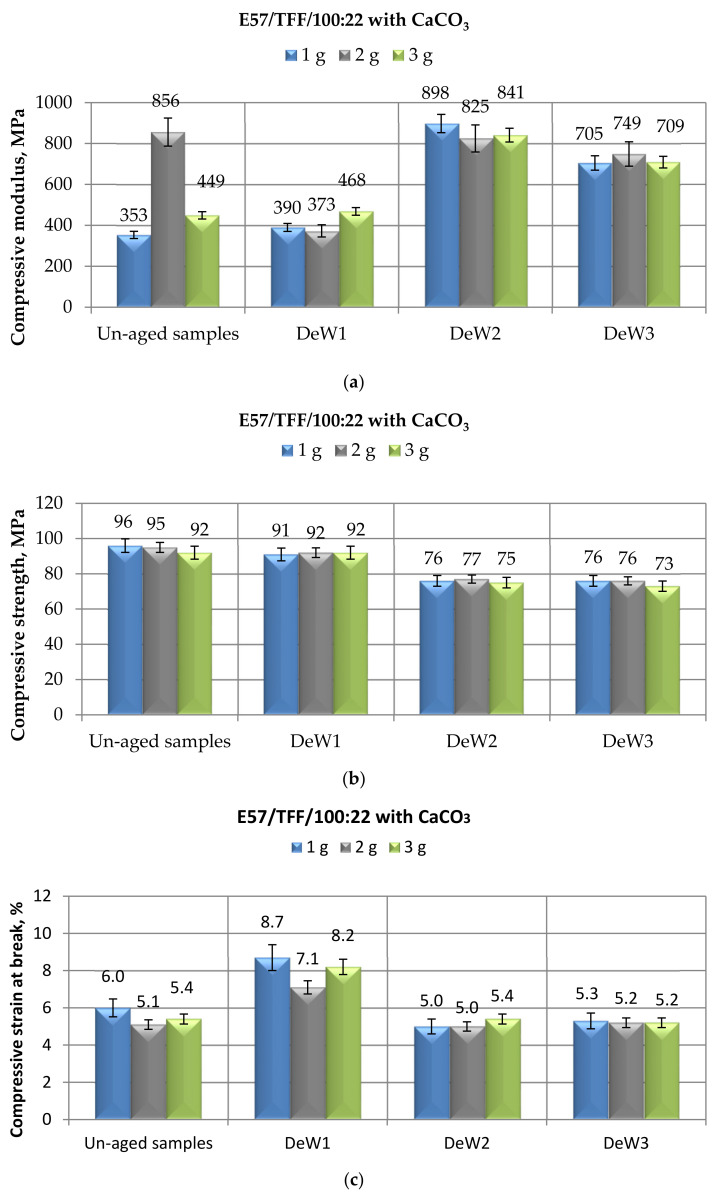
Results of mechanical tests performed in compression mode on CaCO_3_-filled E57/TFF/100:22 specimens after different immersion times (i.e., 1, 6, and 10 months) in demineralized water, in terms of (**a**) Compressive modulus; (**b**) Compressive maximum strength; (**c**) Compressive strain at break.

**Figure 4:**
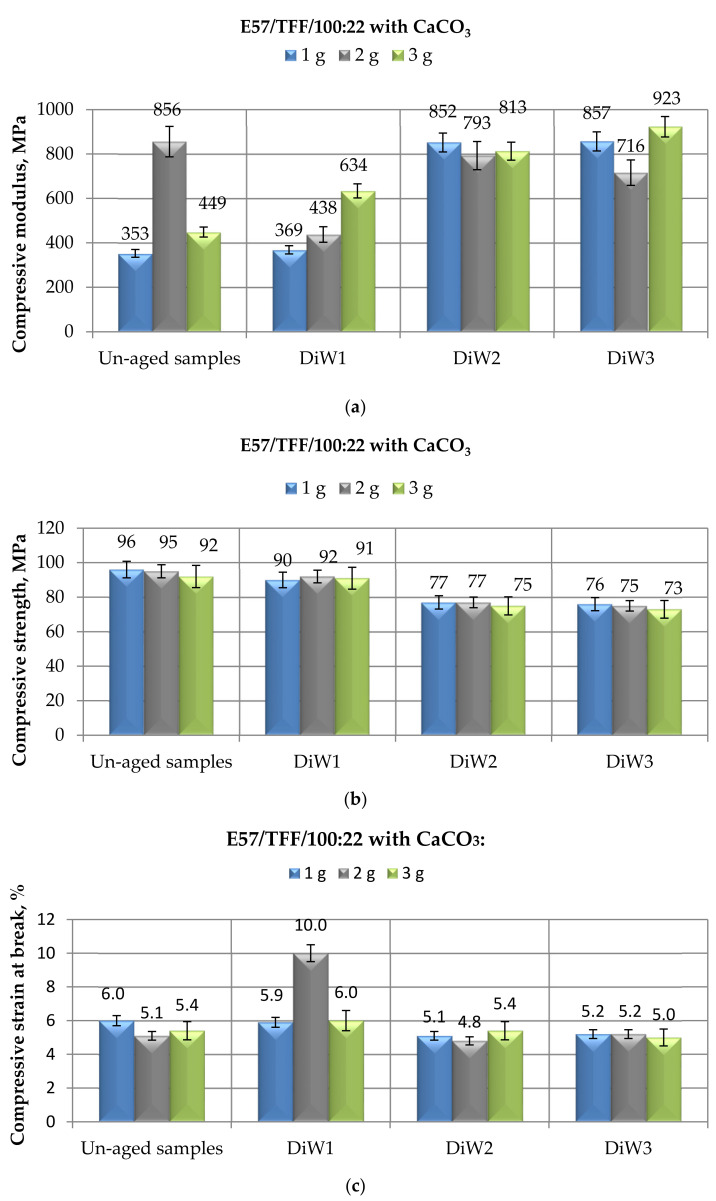
Results of mechanical tests performed in compression mode on CaCO_3_-filled E57/TFF/100:22 specimens after different immersion times (i.e., 1, 6, and 10 months) in distilled water, in terms of (**a**) Compressive modulus; (**b**) Compressive maximum strength; (**c**) Compressive strain at break.

**Figure 5:**
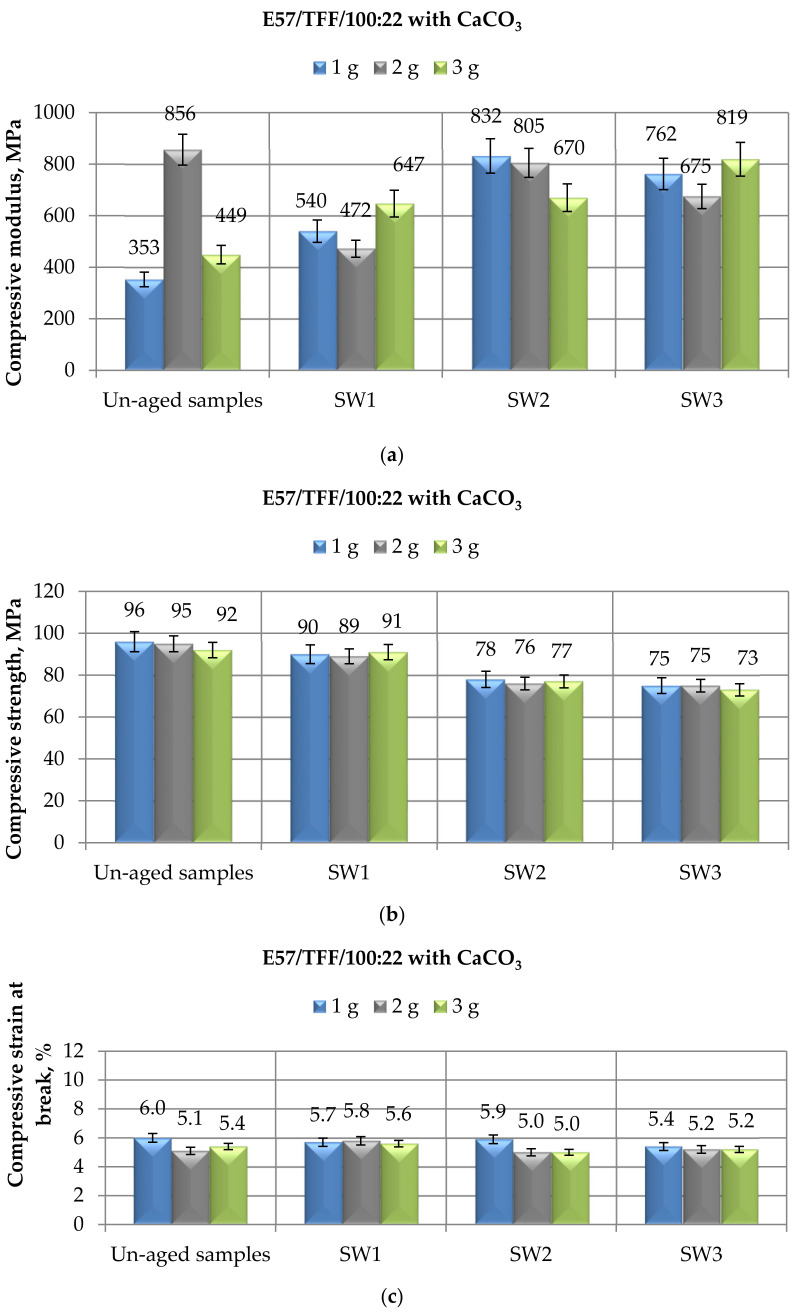
Results of mechanical tests performed in compression mode on CaCO_3_-filled E57/TFF/100:22 specimens after different immersion times (i.e., 1, 6, and 10 months) in spring water, in terms of (**a**) Compressive modulus; (**b**) Compressive maximum strength; (**c**) Compressive strain at break.

Then, the research was devoted to the assessment of the mechanical properties, tested in compression mode, of the epoxy adhesive cold-cured by the Mannich base curing agent and containing different amounts (from 1 to 3 g) of CaCO_3_ micro-filler, after prolonged exposures (up to 10 months) to three selected aqueous environments, reproducing realistic conditions involving immersion in unsalted water. The effects on the compressive mechanical characteristics of E57/TFF/100:22 system filled with calcium carbonate after immersion in demineralized, distilled, and spring water are presented in Figure 3, Figure 4 and Figure 5, respectively. Generally speaking, an increase in the modulus as a consequence of the exposure was observed, especially at longer immersion times, coupled with limited reductions in maximum strength and very small changes in strain at break; the extent of the modifications seems to be influenced by the filler content to a limited extent.

Analyzing in detail the data recorded after different aging times in demineralized water (Figure 3a), this environment produced an appreciable increase in the compressive modulus of the filled epoxy after 6 months of immersion, this property being even more than doubled with respect to the value measured on the unaged compounds. Short times of immersion (i.e., 1 month) affected to a lower extent this property, irrespective of the filler content. After the longest period (10 months) of exposure to demineralized water, a small reduction in modulus was observed; however, this characteristic remained well above the values measured on unaged specimens, irrespective of the filler content and on unfilled epoxy (see Figure 2a). The only exception is represented by the E57/TFF/100:22 system filled with 2 g of calcium carbonate: in this compound, in fact, starting from a much greater value of modulus with respect to the formulations filled with 1 and 3 g of CaCO_3_, this property experienced an appreciable reduction after 1 month of immersion, even if the absolute value of the modulus is comparable to those measured on the other filled systems aged for the same time. Then, at longer immersion times, the modulus increased to a value close to that measured for unaged system. However, as already observed, a large standard deviation value was measured for the result relative to unaged E57/TFF/100:22/2CaCO_3_: this would explain the substantially different behavior of the modulus of this formulation upon immersion in demineralized water. Therefore, these data deserve additional investigation.

After 1 month of exposure, the maximum strength of CaCO_3_-filled Mannich base-cured E 57 epoxy systems experienced very small reductions with respect to the initial values (Figure 3b). However, by increasing the immersion times in demineralized water, almost steady values of this characteristic were measured, with average decreases around 20%, irrespective of the filler content.

The effect of the same environment was minimal on the compressive strain at break of the E57/TFF/100:22 system filled with different amounts of calcium carbonate after 6 and 10 months of exposure, respectively (Figure 3c). On the other hand, the values registered for all the filled systems after 1 month of immersion display an increase around 40–50%, irrespective of the filler content.

The authors should discuss the results and how they can be interpreted from the perspective of previous studies and of the working hypotheses. The findings and their implications should be discussed in the broadest context possible. Future research directions may also be highlighted.

The observed results are substantially in line with those obtained cold-curing CaCO_3_-filled Epidian 5 epoxy with the same curing agent and exposing these systems to demineralized water for the same immersion periods [35]: an increase in compressive modulus, especially for longer (i.e., 6 and 10 months) immersion times and a limited reduction in the maximum strength up to a 6-month immersion time. Referring to the compressive strain, in the previous study, reductions (up to 30–35%) due to the immersion in demineralized water for medium-longer times were observed; however, these were starting from substantially greater values (even doubled if the system was filled with 2 g of calcium carbonate) of this property. These observations allow confirming the positive effect of a cheap calcium carbonate micro-filler against the plasticization of epoxy caused by water.

The investigation continued by analyzing the mechanical behavior of CaCO_3_-filled E 57 epoxy cold-cured by the Mannich base curing agent after a prolonged aging in distilled water, whose results are reported in Figure 4. The trends of modulus (Figure 4a), maximum strength (Figure 4b), and strain at break (Figure 4c) of the filled-epoxy specimens tested in compression mode after different time spans of exposure to distilled water largely reflect those observed when the immersion was performed for the same periods of time in demineralized water (Figure 3).

Noticeable growths in modulus were recorded on the CaCO_3_-filled epoxy at the longer immersion times, i.e., 6 and 10 months (Figure 4a); the same property was somewhat affected by a 1-month immersion period, especially at the lower filler contents (1 and 2 g). However, the value of the modulus of 2 g of unexposed CaCO_3_-filled E57/TFF/100:22 should be substantiated. Apart from this value, it is confirmed that the compressive modulus of the aged filled specimens is always much greater than that of unimmersed specimens as well as of the unfilled E57/TFF/100:22.

The trend and the values of compressive maximum strength measured on CaCO_3_-filled E 57 epoxy exposed to distilled water, as reported in Figure 4b, are fully comparable to those recorded for the same system immersed for the same periods of time in demineralized water, as reported in Figure 3b. Therefore, the same considerations can be repeated in this case. Once again, the filler content seems to have an irrelevant effect on this characteristic, even after a prolonged aging exposure.

The data relative to the strain at break measured on the E57/TFF/100:22 systems filled with different amounts of calcium carbonate and immersed up to 10 months in distilled water are presented in Figure 4c. In this case, an appreciable increase (i.e., +85%) in this characteristic after 1 month of immersion was recorded only for the system filled with 2 g of CaCO_3_ micrometric particles; for the other two filled systems, on the other hand, the compressive strain at break remained substantially unchanged from its initial values. By increasing the exposure times to 6 and 10 months, finally, values of compressive strain very marginally lower than those registered on the unaged specimens were recorded, confirming the data and the trend observed for the same filled systems immersed in demineralized water (Figure 3c).

Once again, when comparing these results with those found on Epidian 5 epoxy filled with the same amounts of calcium carbonate, cold-cured with the same curing agent, and immersed in distilled water for the same periods of time, it is noted that the trends observed in the present study for compressive modulus, maximum strength, and strain at break are basically in line with those previously reported [35]. The only exception is represented by compressive strain variations as a consequence of the immersion in distilled water, which were greater in the previous study: however, we can repeat the same considerations already outlined, i.e., the initial values of this property measured on unaged Epidian 5-based filled systems were considerably greater.

Finally, in Figure 5, the mechanical behavior tested in compression mode of CaCO_3_-filled E57/TFF/100:22 systems aged in spring water up to 10 months is displayed: the compressive modulus (Figure 5a), the maximum strength (Figure 5b), and the strain at break (Figure 5c) can be compared to the values of the same characteristics measured on unaged systems. It can be observed that the results are roughly in line with those found for the same systems aged for the same time spans in demineralized water (Figure 3) and distilled water (Figure 4), respectively.

In this case, the compressive modulus increased more gradually by increasing the immersion times (Figure 5a), achieving the greatest values after 6 or 9 months of immersion in spring water for E57/TFF/100:22 systems filled with 1 g and 3 g of calcium carbonate, respectively. The only exception is again represented by the E57/TFF/100:22/2CaCO_3_ compound, whose initial value (i.e., measured on the unaged specimens) should be confirmed.

As regards the compressive maximum strength data, apart from the inevitable variations caused by the experimental measurements, the numerical data reported in Figure 5b are fully comparable with those reported in Figure 3b and Figure 4b after aging in demineralized water and distilled water, respectively.

The values of compressive strain at break reported for the CaCO_3_-filled E 57 epoxy systems in Figure 5c suggest a substantial constancy of this property upon immersion in spring water, even after a 1-month immersion period. Therefore, it is confirmed that for such filled systems, an aqueous environment has a minimal influence on this parameter.

Finally, the comparison of the results of mechanical tests performed in compression mode on CaCO_3_-filled E57/TFF/100:22 compounds immersed in spring water can be compared to the findings from an analogous investigation carried out on Mannich base-cured Epidian 5 epoxy filled with the same amounts of the same micro-filler and exposed for the same time spans in spring water [35]. The same comments already outlined in the case of specimens exposed to the other aging regimes can be here repeated: the effects of an immersion in spring water on the compressive mechanical properties of the TFF cold-cured CaCO_3_-filled Epidian 5 epoxy measured in the previous study are very similar to what was experienced in the present study, with the exception of the larger reductions measured for compressive strain at break.

As already underlined, a distinctive advantage displayed by epoxy adhesives is represented by the possibility to select the type of resin, curing agent, and filler; the composition of the final system; and the curing conditions (in terms of temperature and time) in order to create the highest performing compound with respect to a specific application. Therefore, referring to an application for which frequent contact with a non-saline aqueous environment is expected, if the application requires the resin to withstand higher load resistance values (constantly greater than 100 MPa), the best choice would be CaCO_3_-filled Epidian 5 resin cold-cured with the Mannich base curing agent. On the other hand, if the adhesive must maintain the strain values substantially unchanged during the application of a compression load during its immersion in an aqueous environment, the choice should probably fall on one of the CaCO_3_-filled E57/TFF/100:22 systems.

### 3.3. Effect of Aqueous Aging Regimes on CaCO_3_-Filled Triethylenetetramine-Cured Epoxy Adhesives

**Figure 6:**
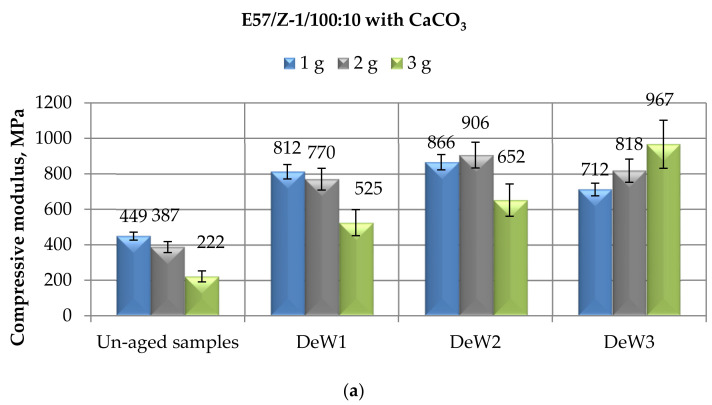

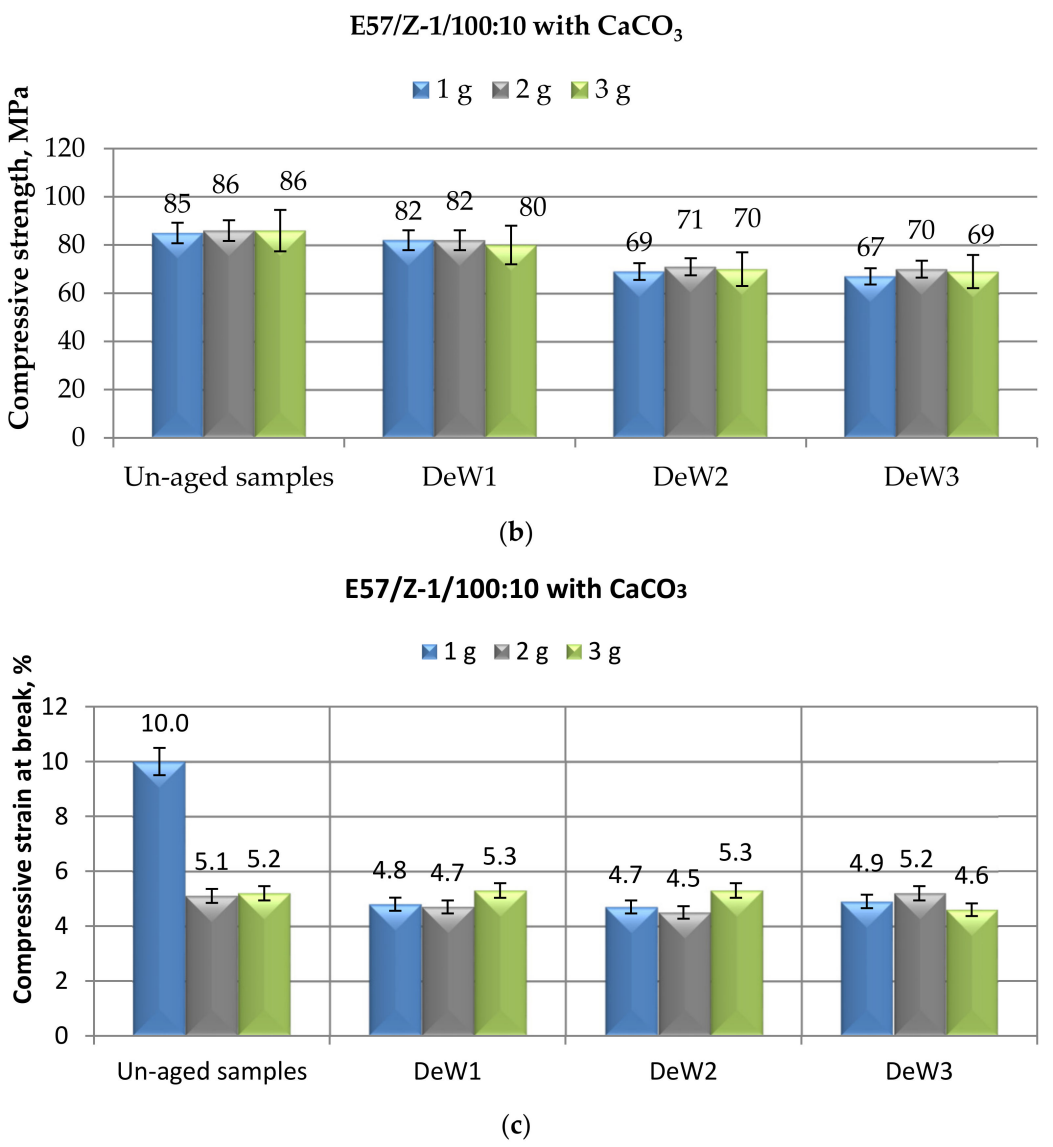
Results of mechanical tests performed in compression mode on CaCO_3_-filled E57/Z-1/100:10 specimens after different immersion times (i.e., 1, 6, and 10 months) in demineralized water, in terms of (**a**) Compressive modulus; (**b**) Compressive maximum strength; (**c**) Compressive strain at break.

**Figure 7:**
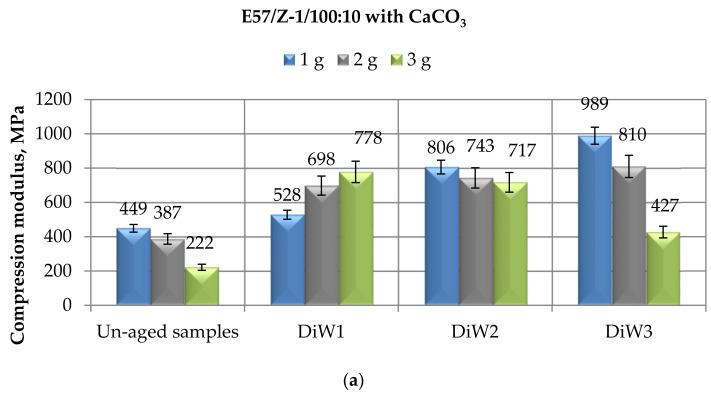

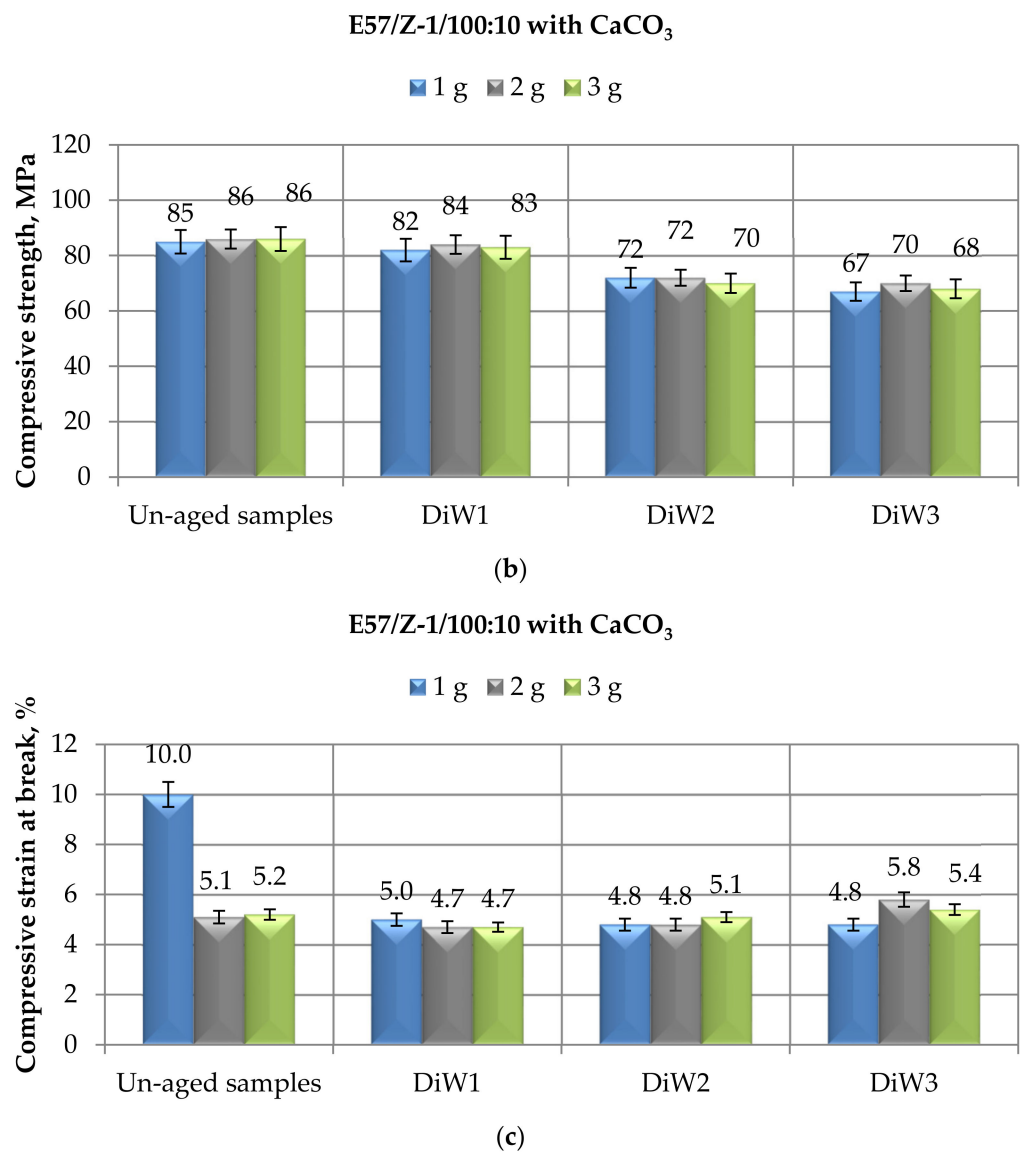
Results of mechanical tests performed in compression mode on CaCO_3_-filled E57/Z-1/100:10 specimens after different immersion times (i.e., 1, 6, and 10 months) in distilled water, in terms of (**a**) Compressive modulus; (**b**) Compressive maximum strength; (**c**) Compressive strain at break.

**Figure 8:**
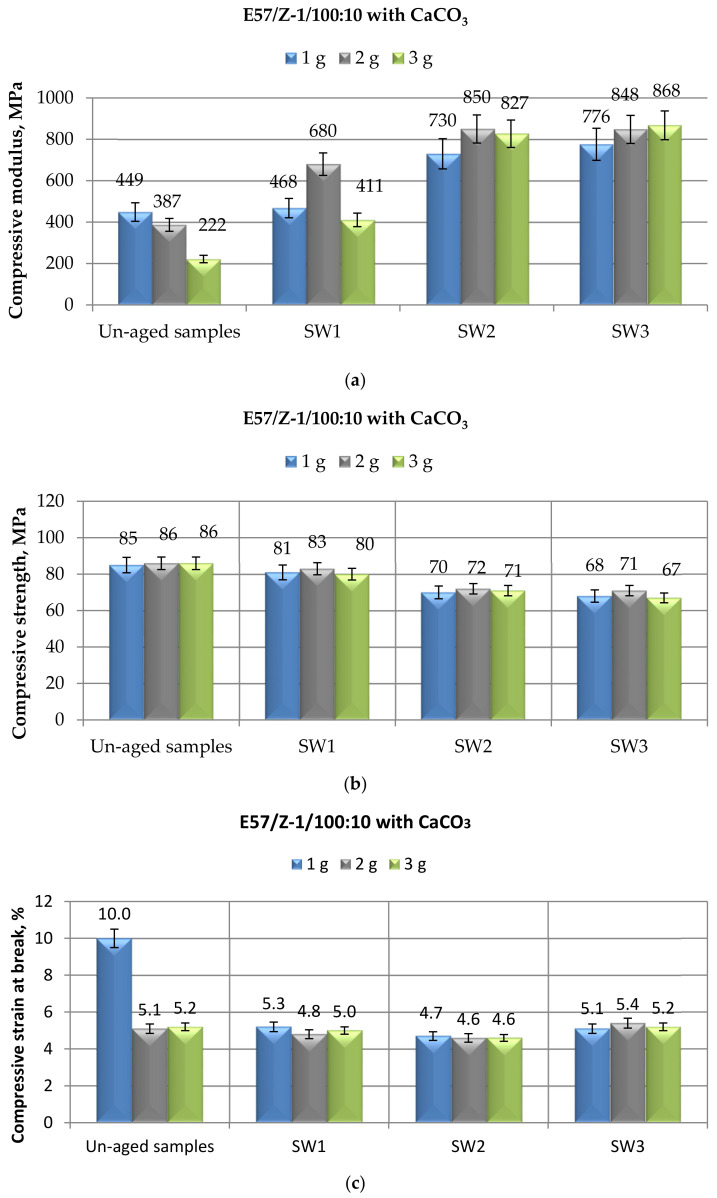
Results of mechanical tests performed in compression mode on CaCO_3_-filled E57/Z-1/100:10 specimens after different immersion times (i.e., 1, 6, and 10 months) in spring water, in terms of (**a**) Compressive modulus; (**b**) Compressive maximum strength; (**c**) Compressive strain at break.

The same investigation performed on the CaCO_3_-filled E57/TFF/100:22 systems, aged in three aqueous environments and illustrated in the previous paragraph, was repeated by cold-curing the same filled epoxy resin, Epidian 57, with the TETA curing agent and subjecting the obtained systems to (compressive) mechanical tests after 1, 6, and 10 months of immersion in demineralized, distilled, and spring water, respectively. The results of these tests in terms of modulus, maximum strength, and strain at break are reported in Figure 6, Figure 7 and Figure 8.

The results recorded for the E57/Z-1/100:10 systems containing different amounts of calcium carbonate and immersed up to 10 months in demineralized water are first introduced (Figure 6). Referring to the compressive modulus (Figure 6a), this property appreciably increased by increasing the immersion time up to 6 months. Upon the longer exposure (i.e., 10 months), it decreased to a small extent, still remaining well above the values measured for the unfilled specimens. In this case, the amount of filler seems to have a certain effect, decreasing the modulus at the greatest filler content (3 g). On the other hand, the value registered for the E57/Z-1/100:10/3CaCO_3_ system seems to be out from this general trend: however, the very high standard deviation measured for this value could explain this discrepancy.

The compressive maximum strength (Figure 6b) experienced reductions as a consequence of the exposure to this aqueous regime: very limited decreases (not greater than 7%) were measured after a 1-month period of immersion, while reductions around 18–20% were measured at longer (6 and 10 months) immersion times.

Substantial constant values of compressive strain at break were found for the CaCO_3_-filled E57/Z-1/100:10 systems after different time spans in demineralized water (Figure 6c). Since in the case of the unaged system containing the smallest amount of filler, i.e., E57/Z-1/100:10/1CaCO_3_, a compressive strain much greater (doubled) than those of the other filled specimens was recorded, strong reductions of this parameter were measured as a consequence of the aqueous exposure. However, since this value appears out from the trend, as already underlined, it should be confirmed by further analysis.

The mechanical data reported in Figure 6 can be compared with those reported in Figure 3 relative to CaCO_3_-filled E57/TFF/100:22 specimens immersed for prolonged times in demineralized water. From the comparison, it can be concluded that the mechanical behavior observed for the filled Epidian 57 resin aged in demineralized water is almost independent on the curing agent used. In fact, this exposure caused appreciable increases in compressive modulus (Figure 3a and Figure 6a), limited reductions (in both cases around 20%) in compressive maximum strength (Figure 3b and Figure 6b), and minimal modifications in compressive strain at break values (Figure 3c and Figure 6c).

Another comparison can be done with the results of the investigation performed on TETA-cured Epidian 5 epoxy filled with the same amounts of CaCO_3_-filler, exposed for the same time spans in demineralized water [35]. Similar conclusions were previously drawn for the other filled-resin cold-cured with the same curing agent; i.e., the immersion in demineralized water caused noticeable increases in compressive modulus and limited reductions in maximum strength values. Once again, starting from greater strain at break values in that case (almost doubled), larger reductions of this parameters were experienced with respect to the present study as a consequence of the immersion in demineralized water. These observations will further confirm that the recorded mechanical behavior as a consequence of the exposure to a non-saline aqueous environment can be considered general of a cold-cured bis-phenolic epoxy resin reinforced with the addition of a CaCO_3_ filler, depending mainly on the chemical nature of the filler and on its interactions with the matrix epoxy.

In Figure 7, the mechanical behavior of CaCO_3_-filled E 57 epoxy specimens, cold-cured using a TETA curing agent after a prolonged aging in distilled water is reported. The general trend of results relative to compressive modulus (Figure 7a), compressive maximum strength (Figure 7b), and compressive strain at break (Figure 7c) of the filled-epoxy compounds largely reproduces what was just observed, i.e., when the immersion of the same systems was performed in demineralized water (Figure 6).

The compressive modulus (Figure 7a) experienced a continuous increase during the aging procedure, in particular for the specimens filled with 1 and 2 g of calcium carbonate. The numerical values are somehow lower than those observed when the immersion was performed in demineralized water (data reported in Figure 6a). Moreover, in the case of immersion in distilled water, the greatest values of modulus were recorded after an immersion period of 10 months. The final values measured at this stage were again much greater than the initial ones. Once again, the values of modulus measured for the E57/Z-1/100:10/3CaCO_3_ system appeared roughly out from the trend registered for the other two formulations, with a rapid noticeable growth after only 1 month of immersion and subsequent reductions for longer times in distilled water, particularly after a 10-month exposure. Therefore, these results deserve further investigation.

In Figure 7b, the data relative to the compressive maximum strength of the CaCO_3_-filled E57/Z-1/100:10 systems after different time spans in distilled water are illustrated. Both the trend and the numerical values of these properties are perfectly comparable with those observable in Figure 6b. Therefore, the same considerations can be repeated: the immersion in distilled water caused very small reductions (about 3–4%) after a 1-month period; more significant reductions, but never exceeding 22%, were recorded at longer (6 and 10 months) exposure times. The filler content has a negligible influence irrespective to the immersion time.

The compressive strain at break values measured on the CaCO_3_-filled E57/Z-1/100:10 systems after different periods of time in distilled water are presented in Figure 7c. This characteristic seemed to be almost unaffected by this aqueous exposure, as already observed in Figure 6c in the case of immersion in demineralized water. The same considerations previously reported for the behavior of E57/Z-1/100:10/1CaCO_3_ system can be here repeated.

Finally, the results of the compressive mechanical tests performed on E57/Z-1/100:10 systems filled with different amounts of calcium carbonate reported in Figure 7 can be compared with the data obtained for the CaCO_3_-filled E57-based compounds, cold-cured with the Mannich base, and exposed for the same time spans to distilled water, as reported in Figure 4. Only differences in absolute numerical values were recorded for the two aged systems, differing merely for the curing agent employed, since the trend is essentially the same for all the mechanical properties: a growth in the modulus, which is greater at medium–longer immersion periods (Figure 4a and Figure 7a); small reductions in maximum strength (Figure 4b and Figure 7b); and almost irrelevant changes for strain at break (Figure 4c and Figure 7c).

The general trend of the compressive modulus and strength results is also comparable to that displayed by the CaCO_3_-filled Epidian 5 epoxy system cold-cured with the same curing agent and immersed in distilled water for the same time spans [35]. Regarding the different behavior observed for the strain at break in the two research experiments, the same considerations previously reported can be here repeated. However, in the present work, the effect of filler content on strain at break is negligible, irrespective of the immersion time, if compared to the previous research [35].

The last part of the study was devoted to analyzing the mechanical behavior in compression mode of E57/Z-1/100:10 epoxy systems, containing from 1 to 3 g of the calcium carbonate, after different immersion periods in spring water. The results recorded for these systems are reported in Figure 8.

As already observed for the other two aqueous environments (Figure 6a and Figure 7a), also the permanence in spring water led to increases in the compressive modulus values (Figure 8a), the greater and longer the immersion time, irrespective to the filler content. Therefore, the growing trend of the modulus as a consequence of exposure to a non-saline aqueous environment is confirmed by these latest experiments.

The maximum strength data collected during the compressive mechanical tests, reported in Figure 8b, on the CaCO_3_-filled E57/Z-1/100:10 systems aged in spring water are perfectly in line with those recorded for the same systems after immersion in demineralized water and distilled water (Figure 6b and Figure 7b): decreases in this parameter not exceeding 7% were measured after a 1-month immersion, while reductions around 17% and 20% were recorded after 6 and 10 months of immersion, respectively. Once again, almost insignificant is the effect of the filler content on this parameter, considering the standard deviation of experimental results.

Immersion in spring water negligibly affects the compressive strain at break (Figure 8c) as already observed in the other investigated aqueous environments (Figure 6c and Figure 7c).

Comparing the mechanical behavior of the same filled resin in different compositions, i.e., CaCO_3_-filled Epidian 57 systems, cured with the two different curing agents, i.e., Mannich base and TETA, and exposed to the same environment, i.e., spring water, whose results are reported in Figure 5 and Figure 8, respectively, it can be concluded that this non-saline aqueous environment affects in the same way and, roughly, to the same extent, taking into account inevitable experimental spreads of results, the filled resin, irrespective to the curing agent employ.

Finally, comparing the mechanical data relative to different bis-phenolic epoxy resins analyzed in the present study (Figure 8) and in a previous investigation [35], cold-cured with the same curing agent (i.e., TETA), filled with the same micro-filler and exposed to the same environment (i.e., spring water), it can be concluded that the effect of this environment is fairly equivalent with regard to compressive modulus and strength; the negligible effect on strain at break found in the present study can be attributed to the lower value measured on the unaged specimens for this parameter with respect to the previous research.

As a final conclusion, the mechanical behavior (measured in compression mode) observed for the cold-cured bis-phenolic CaCO_3_-filled epoxy adhesives after aging in a non-saline aqueous environment (demineralized, distilled, or spring water), i.e., noticeable increases in modulus, small reductions in the strength, and limited variations in the strain, can be regarded as general. There is a certain influence of the exposure time, especially when comparing the properties at the lowest time, i.e., 1 month, with those at medium–long times; the behavior does not depend on the type of water as long as it is not salty; it scarcely depends on the filler content (for such low contents, i.e., up to 3 g of the calcium carbonate per 100 g of the epoxy resin); the variations of the compression mechanical properties depend on the initial value of the property (such as the strain reductions observed for Epidian 5 and Epidian 57 resins).

**Figure 9:**
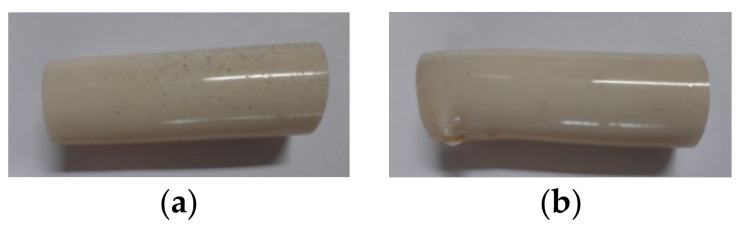

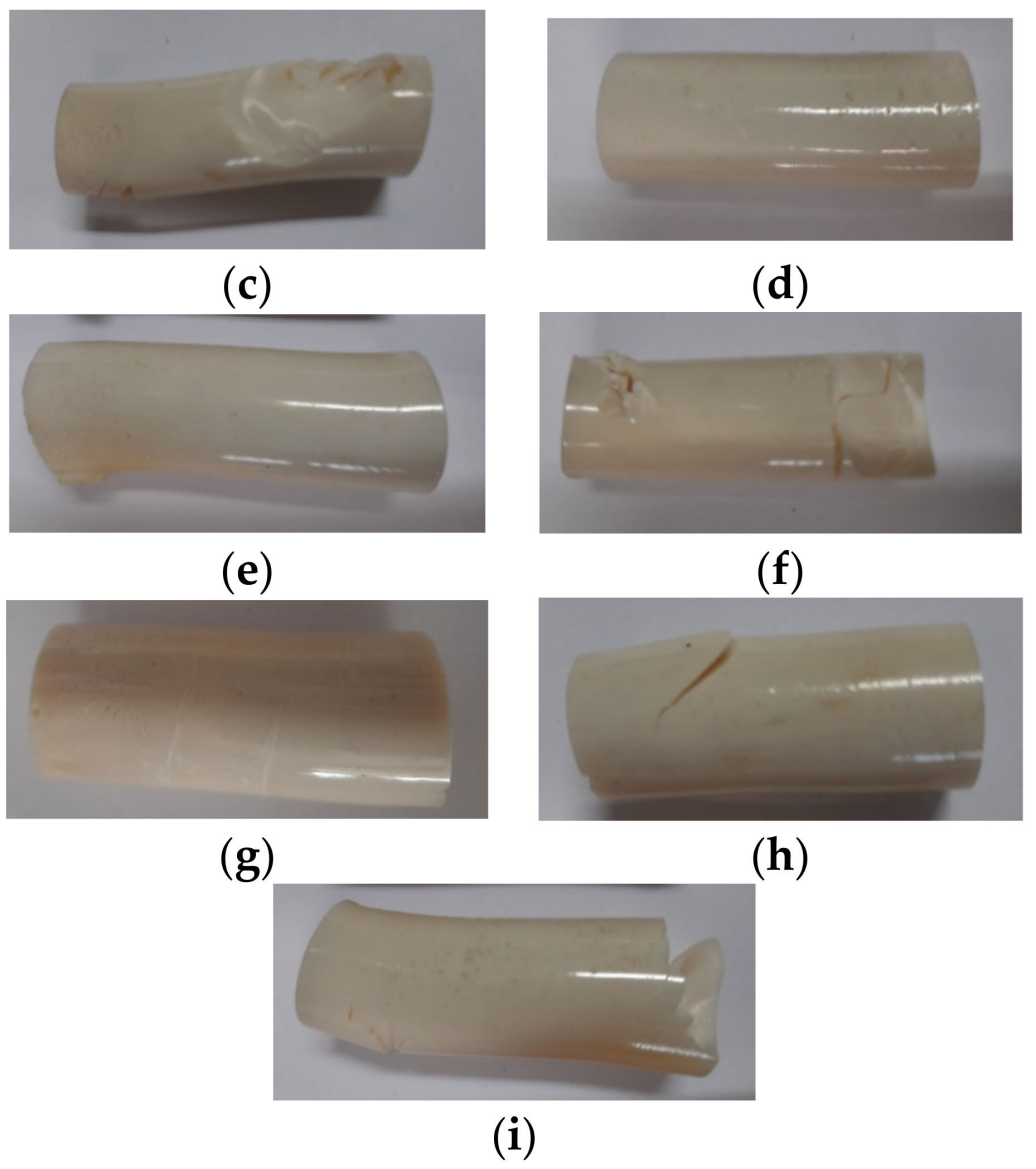
Images of some CaCO_3_-filled epoxy adhesives cold-cured by triethylenetetramine (TETA) curing agent (i.e., system E57/Z-1/100:10/1CaCO_3_) after mechanical (in compression mode) tests: (**a**) DeW1; (**b**) DeW2; (**c**) DeW3; (**d**) DiW1; (**e**) DiW2; (**f**) DiW3; (**g**) SW1; (**h**) SW2; (**i**) SW3.

Finally, in Figure 9, the images (taken with a digital camera) of some of the CaCO_3_-filled E57/Z-1/100:10 specimens fractured during the mechanical tests are reported. The simple visual observation of the pictures revealed that the kind of damage experienced by the CaCO_3_-filled specimens mainly depends on the aging duration, while it apparently was not affected by the aqueous environment, thus further confirming what was revealed by the analysis of the experimental results. From the visual observation of the other compounds, i.e., those containing 2 and 3 g of the calcium carbonate filler, it was also possible to generalize that the time of exposure played a significant role on the kind of failure of the filled adhesives. In particular, it was noted that with increasing the aging time, the permanent deformation became more evident, especially after 6 months of exposure. At the longest aging time, i.e., 10 months, a cohesive failure of the micro-filled adhesive was generally recorded, with a clear appearance of some cracks and spalling.

### 3.4. Discussion

The investigation performed in the present study aimed at assessing how the mechanical behavior, tested in compression mode, of a commercial epoxy reinforced with the addition of small amounts of the CaCO_3_-filler, cold-cured with two different curing agents, i.e., a Mannich base and a triethylenetetramine, both suitable to cure the resin at ambient temperature, can be altered as a consequence of the exposure to an aqueous environment for prolonged times. The findings found in the present study, summarized at the end of the previous paragraph, are discussed, also taking inspiration from the available literature.

As already underlined, the exposure to liquid water led to severe modifications of the properties and performance of epoxy resins. The addition of a proper filler to the resin can limit to a certain extent the detrimental plasticization effects caused by water ingress. When the mechanical properties of an unfilled epoxy are measured in tensile mode, both the elastic modulus and the maximum strength are reduced upon immersion in pure water; the addition of a CaCO_3_ filler to the epoxy resin has been shown to be effective in limiting the reductions in elastic modulus [31,33,34]. The lower reductions in tensile modulus were ascribed to the ability of this filler to hamper the elastic deformation of the epoxy [31,33]. On the other hand, in the case of the tensile strength, the contrast effect of the filler is limited by the fact that absorbed water can also negatively affect the interface matrix/filler, further reducing the tensile resistance [31,33]. This hypothesis would be confirmed by the results of the present study: since the specimens were subjected to a different (compressive) load, reduction in the strength never exceeding 22% was recorded. Bearing in mind the observation just made, which concerns the different load applied in the present study compared to what is reported in the literature that can eventually produce different mechanisms in the presence of the filler, another process may have occurred in the cold-cured resins during their immersion in freshwater, i.e., the continuation of the curing reactions. It is well known that cold-cured epoxy resins take much longer times than those suggested by manufacturers to reach a degree of cross-linking (curing) approaching 100% (months or even years), the recovery of the cure being favored by the decrease in the glass transition temperature, Tg, due to plasticization upon immersion in water [27,28,40]: an increase in curing degree can, at least partly, counteract the detrimental plasticization effects on modulus and strength, as already observed when a commercial cold-cured epoxy adhesive immersed in demineralized water for longer times was tested in tensile mode [41].

The exposure to an aqueous aging of epoxies added with a CaCO_3_ filler resulted in the growth of their tensile strain at break due to an increased ductile behavior caused by plasticization [31,33,34]. As already underlined, the variations of this property due to the exposure to water can be to some extent inhibited by the presence of the CaCO_3_ micro-filler, which is able to hinder the absorption of the liquid in the resin [31]. Furthermore, the irregular shape of the CaCO_3_ particles was found to additionally contribute to reduce the deformability of the resin [31]. On the other hand, the mechanisms taking place in the epoxy resin when applying a tensile or compressive load are likely to be very different: therefore, the effect of aging in water on the tensile or compressive characteristics can be very different.

### 3.5. Statistical Analysis of Compressive Modulus Results

The analysis of the compressive modulus data, for which a greater spread of results was observed, was performed according to the ANOVA of the main effects, i.e., the MANOVA analysis. This is a multivariate analysis of variance used to assess the impact of different factors on the variable under study. This method was chosen since the variable under study, i.e., the modulus measured in compression mode, is influenced by several factors (type of the curing agent, the amount of calcium carbonate filler, type of the aqueous environment, and time of aging): therefore, it is important to analyze the influence of each factor on this variable.

For the purpose of this statistical analysis:

- The adjustment of the results of empirical studies of the analyzed variables to the normal distribution was verified using the Shapiro–Wilk normality test (S-W);
- The distribution of the results obtained in the analyzed test variants was assessed, being consistent with the normal distribution.
- Verification of the assumption of homogeneity of variance was performed using Levene’s test.

On the basis of the obtained results, the assumption of the normality of the distribution and homogeneity of variance was fulfilled (at a significance level α = 0.05). Then, the MANOVA analysis was performed for compressive modulus data collected for unaged and immersed specimens.

The results of the MANOVA statistical analysis applied to compressive modulus data allow concluding the following:

- With the probability level of *p* = 0.86644, there is no basis to reject the hypothesis that the “Type of aqueous environment” factor has no effect on compressive modulus;
- With the probability level of *p* = 0.000000, the hypothesis about no effect of the “Aging duration” factor should be rejected;
- With the probability level of *p* = 0.000000, the hypothesis that the “Amount of filler” factor has no effect should be rejected;
- With the probability level of *p* = 0.098346, there is no basis to reject the hypothesis of the lack of effect of the “Type of curing agent” factor.

Therefore, when considering the compressive modulus data collected in the present study for CaCO_3_-filled epoxy adhesives, before and after their exposure to the three selected aqueous aging regimes, the analysis of variance demonstrated that both the aging duration and the amount of filler have a significant impact on the value of this mechanical characteristic; on the other hand, the type of aqueous environment did not significantly affect the modulus. Finally, this statistical analysis demonstrated that the type of curing agent influenced compressive modulus to a very limited extent.

An appropriate post-hoc test was used to compare statistically significant factors. To this aim, the HSD Tukey test (“honestly significant difference” or “honest significant difference”) was employed, since it enables a homogeneous grouping of data with indication of significant differences. The Tukey test is used to determine if the relationship between two sets of data is statistically significant—that is, whether there is a strong chance that an observed numerical change in one value is causally related to an observed change in another value. The post-hoc test, the HSD Tukey test of homogeneous groups and significant differences, was performed. The obtained results are summarized in Table 5, Table 6, Table 7 and Table 8 for aging duration and for the amount of filler, respectively.

When analyzing the obtained results, it can be noticed that Tukey’s test separated three homogeneous groups. The samples aged for 6 and 10 months are in one homogeneous group, the remaining data belong to separate groups. This result would suggest that there are no significant differences between the samples aged for 6 months and for 10 months at the assumed significance level of α = 0.05. The obtained results were also confirmed by Tukey’s significant differences test: significant differences were not found between the groups of samples aged for 6 and 10 months, the obtained p values (i.e., those marked in black in the table) are greater than the adopted one, i.e., 0.05.

When the compressive modulus data were grouped according to the amount of filler, Tukey’s test distinguished two homogeneous groups. The modulus of pristine (unfilled) epoxy compounds was significantly different from the same property measured on the filled epoxy compounds, which was also confirmed by Tukey’s test of significant differences. The closer the value in this table (shown in black) to 1, i.e., it is not significantly different, the closer the results in these groups are to each other.

The obtained results quantitatively confirmed what was already observed during the analysis of the results of compressive tests performed on calcium carbonate-filled epoxy compounds cured with two different curing agents and exposed for different times to different aqueous regimes.

On the basis of the presented MANOVA statistical analysis, it should be emphasized that among the compositional factors (i.e., the type of curing agent employed to cure the epoxy compounds and the micro-filler content), only the amount of CaCO_3_ filler significantly affects the compressive modulus. The reproduced aging regimes, on the other hand, did not affect at all the compressive modulus, while the durability tests durations noticeably did.

## 4. Conclusions

The results of this study allowed confirming what was already been observed in a previous work, namely the effectiveness of adding small quantities of an inorganic inexpensive macro-filler, such as a calcium carbonate, to counteract the detrimental effects of aging in water on compressive properties of cold-curing epoxy adhesives. It was observed that with the addition of low amounts (from 1 to 3 g per 100 g of resin) of the CaCO_3_ filler, the epoxy adhesive, cured with two different curing agents suitable for the cure at ambient temperature, experienced noticeable increases in modulus upon immersion in different non-saline aqueous environments; the strain was somehow reduced, while the compressive strength was only barely affected by these aging regimes. The effect of the kind of the non-saline aqueous environment analyzed (demineralized, distilled, or spring water) was substantially negligible, as also confirmed by the MANOVA statistical analysis performed on the compressive modulus results. Referring to the exposure times, the mechanical characteristics were affected by this parameter only when comparing the properties at the lowest immersion time with those at medium-long times.

Analyzing the compressive mechanical properties relative to the unaged filled and the pristine epoxy adhesives, it was concluded that the micro-filler was able to affect to a great extent the compressive modulus, which increased with respect to the unfilled resin, and the strain values, which experienced noticeable reductions, irrespective to the curing agent used; once again, the strength of the epoxy was scarcely influenced by the addition of the filler. The mechanical characterization showed that the filler content had a marginal effect on the results. In any case, the CaCO_3_-filled epoxy, even after the exposure to an aqueous environment, was able to maintain the modulus much greater than, and the maximum strength very close to, the same characteristics measured on the unaged control adhesives.

## Figures and Tables

**Table 1:** Properties and characteristics of Epidian 57 epoxy resin.

Property, Characteristic	Epidian 57
Viscosity at 20 °C (mPa·s)	13,000–19,000
Density at 20 °C (g/cm^3^)	1.14–1.17
Curing time at 25 °C suggested by suppliers (days)	7–14
Physical state and color	Viscous yellow-light brown liquid

**Table 2:** Properties and characteristics of curing agents.

Property, Characteristic	Curing Agent	
	TFF	Z-1
Viscosity at 25 °C (mPa·s)	max 10,000	25–30
Density at 20 °C (g/cm^3^)	1.15–1.20	0.978–0.983
Physical state and color	Thick light brown liquid	Yellow-light green liquid

**Table 3:** Compositions of epoxy compounds produced with and without calcium carbonate filler.

Epoxy Compound Composition	Resin/Curing Agent (w/t)	Amount of CaCO_3_ per 100 g Resin (g)
E57/TFF/100:22/1CaCO_3_	100:22	1 g
E57/TFF/100:22/2CaCO_3_		2 g
E57/TFF/100:22/3CaCO_3_		3 g
E57/Z-1/100:10/1CaCO_3_	100:10	1 g
E57/Z-1/100:10/2CaCO_3_		2 g
E57/Z-1/100:10/3CaCO_3_		3 g

**Table 4:** Aging regimens.

Aqueous Environment	Aging Duration (Months)	Aging Code
Demineralized water	1	DeW1
	6	DeW2
	10	DeW3
Distilled water	1	DiW1
	6	DiW2
	10	DiW3
Spring water	1	SW1
	6	SW2
	10	SW3

**Table 5:** Homogeneous groups isolated in the Tukey post-hoc test for aging duration.

Subclass No	HSD Tukey Test, Variable: Compression Modulus [MPa] Homogeneous Groups, Alpha = 0.05000 Error: Intergroup MS = 34952, df = 327.00				
	Storage Time	Compression Modulus, MPa	1	2	3
4	Lack	339.3800	–	****^1^	–
1	1 month	560.5699	–	–	****^1^
2	6 months	790.1600	****^1^	–	–
3	10 months	806.6699	****^1^	–	–

^1^ ****—indicates the presence of a homogeneous group.

**Table 6:** Significant differences between the results sorted by aging duration.

Subclass No	HSD Tukey Test, Variable: Compression Modulus [MPa] Approximate Probabilities for Post Hoc Testing Error: Intergroup MS = 34952, df = 327.00				
	Storage Time	{1} 560.57	{2} 790.16	{3} 806.67	{4} 339.38
1	1 month	–	0.000008	0.000008	0.000008
2	6 months	0.000008	–	0.922753	0.000008
3	10 months	0.000008	0.922753	–	0.000008
4	Lack	0.000008	0.000008	0.000008	–

**Table 7:** Homogeneous groups isolated in the Tukey post-hoc test for the amount of filler.

Subclass No	HSD Tukey Test, Variable: Compression Modulus [MPa] Homogeneous Groups, Alpha = 0.05000 Error: Intergroup MS = 47392, df = 327.00			
	Amount of Filler	Compression Modulus, MPa	1	2
1	0	50.3300	–	****^1^
4	3	684.8426	****^1^	–
2	1	710.5044	****^1^	–
3	2	713.6100	****^1^	–

^1^ ****—indicates the presence of a homogeneous group.

**Table 8:** Significant differences between the results sorted by amount of filler.

Subclass No	HSD Tukey test, Variable: Compression modulus [MPa] Approximate probabilities for post hoc testing Error: intergroup MS = 34952, df = 327.00				
	Amount of Filler	{1} 50.330	{2} 710.50	{3} 713.61	{4} 684.84
1	0	–	0.000008	0.000008	0.000008
2	1	0.000008	–	0.999599	0.817330
3	2	0.000008	0.999599	–	0.776532
4	3	0.000008	0.817330	0.776532	–

## Data Availability

Data sharing not applicable.
